# Comparison of anterior segment changes after femtosecond laser LASIK and SMILE using a dual rotating Scheimpflug analyzer

**DOI:** 10.1186/s12886-019-1257-0

**Published:** 2019-12-11

**Authors:** Bu Ki Kim, Su Joung Mun, Young Hoon Yang, Ji Sun Kim, Jun Hyung Moon, Young Taek Chung

**Affiliations:** 1 Onnuri Smile Eye Clinic, Hyobong building 9F 1, Gangnam-daero 65 gil, Seocho-gu, Seoul, Republic of Korea; 2 Onnuri Eye Hospital, 325, Baekje-daero, Wansan-gu, Jeonju-si, Jeollabuk-do Republic of Korea

**Keywords:** Small incision lenticule extraction, SMILE, LASIK, FS-LASIK, Galilei, Dual rotating Scheimpflug analyzer

## Abstract

**Background:**

To compare the changes in the anterior segment after femtosecond laser in situ keratomileusis (FS-LASIK) and small incision lenticule extraction (SMILE) using a dual rotating Scheimpflug (DRS) analyzer (Galilei®; Ziemer Ophthalmology, Port, Switzerland).

**Methods:**

A total of 218 eyes of 109 patients who underwent FS-LASIK or SMILE for myopic correction were retrospectively studied. Ninety-eight eyes of 49 patients who underwent FS-LASIK were compared to 120 eyes of 60 patients treated with SMILE. A DRS analyzer was used for preoperative and 6-month postoperative anterior segment analyses. Measured variables included the central corneal thickness (CCT), anterior chamber depth (ACD), anterior and posterior keratometry (K), anterior and posterior best-fit sphere radius, and maximum posterior elevation (MPE).

**Results:**

After the procedure, the amount of CCT decrease was higher in the SMILE group than in the FS-LASIK group, but it was not statistically significant. The MPE was significantly increased after both procedures (*p* < 0.001 and *p* = 0.001 in the FS-LASIK and SMILE groups, respectively), with the amount of elevation being higher after FS-LASIK than after SMILE even though it was not statistically significant. And there was a significant change in the steep and average posterior K in the FS-LASIK group (*p* = 0.006 and 0.001, respectively), but not in the SMILE group.

**Conclusions:**

Regarding changes in the MPE and posterior K, changes in the posterior corneal surface were greater after FS-LASIK than after SMILE.

**Trial registration:**

The trial registration number: KCT0003628. Date of registration: 15 March 2019.

## Background

Laser in situ keratomileusis (LASIK) is the most commonly used type of corneal refractive surgery; however, there are limitations associated with this procedure, such as flap-related complications and dry eye [1, 2]. Small incision lenticule extraction (SMILE) is a relatively new technique for correcting myopia and myopic astigmatism, involving cutting of the intrastromal lenticule using a femtosecond (FS) laser, followed by manual extraction through a peripheral corneal tunnel incision [3]. This procedure is widely used because of its good predictability, safety, and patient satisfaction [4, 5]. SMILE is thought to be biomechanically better than LASIK because it preserves the anterior segment of the cornea, which has greater biomechanical strength than the posterior segment [6, 7].

Accurate analysis of the cornea is important for preoperative risk assessment and early detection of postrefractive surgery ectasia [8]. The DRS analyzer (Galilei®; Ziemer Ophthalmology, Port, Switzerland) uses two rotating Scheimpflug cameras in combination with a Placido topography system. It uses a Placido disc to yield more accurate anterior curvature topographic data, in addition to the data obtained from the Scheimpflug cameras [9]. Moreover, the dual system obtains images from both sides, which minimizes the effect of decentration due to eye movement on corneal pachymetry and posterior corneal curvature measurements [10]. Overall, the DRS analyzer provides anterior segment measurements with good repeatability and reproducibility for both normal and postrefractive surgery corneas [11].

There have been numerous studies comparing the clinical outcomes of LASIK and SMILE, but, to the best of our knowledge, no previous study has compared the anterior segment morphological parameters after FS-LASIK and SMILE using a DRS analyzer. In the present study, we used a DRS analyzer to evaluate changes in the anterior segment following FS-LASIK (Visumax® & Mel 90®; Carl Zeiss Meditec, Jena, Germany) and SMILE (Visumax®; Carl Zeiss Meditec) for myopic correction.

## Methods

### Patients

We conducted a retrospective analysis of 218 eyes (109 patients) that underwent FS-LASIK or SMILE at the Onnuri Smile Eye Clinic, Seoul, Republic of Korea between July 2015 and April 2017. Of these eyes, 98 (49 patients) underwent FS-LASIK and 120 (60 patients) underwent SMILE. Inclusion criteria for the study were as follows: myopia up to − 6.0 diopters (D), astigmatism up to − 3.0 D, minimum age of 18 years, corrected distance visual acuity (CDVA) of 20/40 or better, and a minimum calculated postoperative residual bed thickness (RBT) of 300 μm. Exclusion criteria were as follows: active or residual ocular disease, a prior history of ocular surgery, immunocompromised, or status of pregnant or nursing. The study was approved by the Public Internal Regulatory Board of the Ministry of Health and Welfare, Korea (P01–201802–21-002). All procedures adhered to the tenets of the Declaration of Helsinki, and written informed consent for study participation was obtained from all participants.

Patients underwent a preoperative examination that included measurement of their CDVA, manifest and cycloplegic refraction, and intraocular pressure via tonometry (CT-80®; Topcon, Tokyo, Japan). Patients also underwent slit-lamp microscopy examination, fundus examination, autokeratometry (KR-8900®; Topcon), specular microscopy (noncom Robo-ca®; Konan Medical, Hyogo, Japan), and DRS analyses. Central corneal thickness (CCT) was defined as the thinnest corneal thickness using the DRS analyzer [12]. The ablation depth (AD) value was exported from the Mel 90® program in FS-LASIK, and the lenticule thickness (LT) was exported from the Visumax® program in SMILE. The estimated RBT was obtained by subtracting the cap or flap thickness and AD or LT from the preoperative CCT.

### Galilei dual rotating Scheimpflug analyzer

All eyes underwent corneal topographic analyses using a Galilei® G4 DRS analyzer (software version 6.1.4), preoperatively and at 6 months after SMILE. Measurements with the DRS analyzer were performed according to the manufacturer’s guidelines. All measurements were performed between 10 AM and 3 PM with non-dilated pupils under identical lighting conditions. The device was brought into focus and the patient’s eye was aligned along the visual axis by a central fixation light. The patients were then asked to blink completely just before the measurement. Measured variables included the CCT, anterior chamber depth (ACD), anterior and posterior keratometry (K), anterior and posterior best-fit sphere (BFS) radius, and maximum posterior elevation (MPE). The BFS was determined using the central 8.0-mm zone of the preoperative cornea, which was identical for both the preoperative and postoperative maps. The change in MPE was defined by the maximum forward protrusion of the posterior cornea above the BFS in the 4.0-mm zone. The MPE was calculated by subtracting the preoperative elevation data from the postoperative elevation data based on the maximum difference in the central 4.0-mm zone. Forward protrusion of the posterior cornea was recorded as a negative number.

### Surgical procedures

All surgeries were performed by two experienced ophthalmologists (CYT and KBK), and the target refraction was emmetropia in all eyes.

FS-LASIK was performed using a Visumax® FS laser for flap creation, followed by a Mel 90® excimer laser for stromal ablation. FS-LASIK was performed with topical anesthesia using 0.5% proparacaine hydrochloride (Alcaine®; Alcon, Fort Worth, TX, USA) applied 2–3 min before the procedure. The flap had a 9.0-mm diameter, 110-μm thickness, and a hinge position at the superior position. The optic zone diameter was 6.0~6.5 mm. The target refractive error treatment was applied using an excimer laser after manual lifting of the flap. The stromal bed was washed using a balanced salt solution and the flap was gently repositioned over the stromal bed. A sponge was used to sweep gently radially along the flap edge.

SMILE was performed using a Visumax® FS laser with a repetition rate of 500 kHz, pulse energy of 140 nJ, and spot spacing of 3.5 μm. SMILE was performed with topical anesthesia using 0.5% proparacaine hydrochloride applied 2–3 min before the procedure. The lenticule diameter was 6.0~6.8 mm and the cap diameter was 0.8 mm larger than the lenticule diameter. The intended thickness of the cap was 120 μm, and the incision was 2.0 mm long at the 11 o’clock position. The lenticule was separated with a blunt spatula using Chung’s swing technique, as described previously [13].

After the procedure, 0.5% moxifloxacin (Vigamox®; Alcon) was applied four times daily for 5 days, 0.1% fluorometholone (Opti-V®; Reyon Pharmaceutical, Seoul, Republic of Korea) was used six times daily in the first postoperative 5 days and was gradually tapered for 4 weeks, and preservative-free hyaluronic acid lubricating drops (Tearinfree®; DHP Korea, Seoul, Republic of Korea) were applied for at least 4 weeks.

### Statistical analysis

Statistical analyses were performed using SPSS for Windows statistical software (ver. 15.0; SPSS Inc., Chicago, IL, USA) and the graphs were drawn using Microsoft Excel 2016 software (Microsoft Corp., Redmond, WA, USA). Paired *t-*tests were used to identify differences between pre- and postoperative values. The independent *t-*test was used to compare differences between the two groups. Pearson’s correlation analysis was used to evaluate the relationships between variables. A value of *p* < 0.05 was considered to indicate a statistically significant difference, and the Bonferroni correction was used to account for multiple comparisons [14], for a value of *p* < 0.01 (= 0.05/5).

## Results

Table 1 shows the preoperative data of the two groups. There was no significant difference between the two groups, except in the estimated AD, LT, and RBT (*p* < 0.001). Table 2 summarizes the preoperative and postoperative DRS analyzer parameters of the two groups, which showed no significant difference. Table 3 summarizes the preoperative and postoperative DRS analyzer parameters for the FS-LASIK and SMILE groups. There were significant changes in the ACD, anterior K, MPE, and anterior BFS radius for both groups, and in the steep and average posterior K for the FS-LASIK group after treatments. Table 4 summarizes the pre- and postoperative DRS analyzer parameter differences of the FS-LASIK and SMILE groups. Changes in the ACD showed statistically significant differences between the two groups (*p* < 0.001).

**Table 1:** Preoperative characteristics by group

	FS-LASIK (*n* = 98)	SMILE (*n* = 120)	*p* value
Age (years)	28.00 ± 6.64	27.63 ± 6.63	0.358
Sex (n)			
Male	48	62	N/A
Female	50	58	
Sphere (diopter)	−3.50 ± 1.75	−3.39 ± 1.47	0.809
Cylinder (diopter)	−1.01 ± 0.69	−0.79 ± 0.61	0.071
SE (diopter)	−4.01 ± 1.69	−3.79 ± 1.47	0.827
CCT (*µ*m)	573.17 ± 25.45	575.09 ± 28.84	0.618
Estimated AD/LT (*µ*m)	69.23 ± 22.24	87.20 ± 22.87	< 0.001
Estimated RBT (*µ*m)	393.95 ± 30.16	367.87 ± 22.87	< 0.001

*FS-LASIK* femtosecond laser-assisted laser in situ keratomileusis, *SMILE* small incision lenticule extraction, *SE* spherical equivalent, *CCT* central corneal thickness, *AD* ablation depth, *LT* lenticule thickness, *RBT* residual bed thickness

**Table 2:** Comparison of the preoperative and postoperative DRS analyzer results between the FS-LASIK and SMILE groups

	Preoperative			Postoperative		
	FS-LASIK	SMILE	*p* value	FS-LASIK	SMILE	*p* value
CCT (*µ*m)	573.17 ± 25.45	575.09 ± 28.84	0.618	512.31 ± 31.76	508.56 ± 27.38	0.367
ACD (mm)	3.29 ± 0.26	3.30 ± 0.24	0.728	3.26 ± 0.26	3.23 ± 0.25	0.385
Average K (D)						
Anterior	42.97 ± 1.51	42.71 ± 1.43	0.196	40.28 ± 1.87	39.91 ± 1.67	0.127
Posterior	− 6.18 ± 0.25	− 6.13 ± 0.23	0.128	−6.22 ± 0.26	−6.16 ± 0.23	0.112
Anterior K (D)						
Flat	42.06 ± 1.52	42.00 ± 1.42	0.785	39.61 ± 1.91	39.25 ± 1.69	0.157
Steep	43.89 ± 1.60	43.41 ± 1.53	0.090	40.96 ± 1.86	40.56 ± 1.72	0.116
Posterior K (D)						
Flat	−5.98 ± 0.24	−5.97 ± 0.22	0.630	−6.01 ± 0.24	−5.96 ± 0.23	0.131
Steep	− 6.38 ± 0.28	−6.23 ± 1.17	0.103	−6.43 ± 0.30	−6.37 ± 0.27	0.142
MPE (μm)	7.46 ± 2.59	7.11 ± 2.61	0.330	8.56 ± 2.97	7.86 ± 2.78	0.080
BFSR (mm)						
Anterior	7.88 ± 0.27	7.92 ± 0.25	0.282	8.33 ± 0.34	8.43 ± 0.31	0.128
Posterior	6.54 ± 0.25	6.57 ± 0.23	0.266	6.53 ± 0.26	6.54 ± 0.25	0.742

*DRS* dual rotating Scheimpflug, *FS-LASIK* femtosecond laser-assisted laser in situ keratomileusis, *SMILE* small incision lenticule extraction, *CCT* central corneal thicknes, *ACD* anterior chamber depth, *K* keratometric power, *D* diopters, *MPE* maximal posterior elevation, *BFSR* best-fit sphere radius

*Significant at *p* = 0.01 (=0.05/5), using a Bonferroni adjustment to control for the 5 tests conducted

**Table 3:** Comparison between the preoperative and postoperative DRS analyzer results for the FS-LASIK and SMILE groups

	FS-LASIK			SMILE		
	Preoperative	Postoperative	*p* value	Preoperative	Postoperative	*p* value
ACD (mm)	3.29 ± 0.26	3.26 ± 0.26	< 0.001	3.30 ± 0.24	3.23 ± 0.25	< 0.001
Average K (D)						
Anterior	42.97 ± 1.51	40.28 ± 1.87	< 0.001	42.71 ± 1.43	39.91 ± 1.67	< 0.001
Posterior	−6.18 ± 0.25	−6.22 ± 0.26	0.001	−6.13 ± 0.23	−6.16 ± 0.23	0.104
Anterior K (D)						
Flat	42.06 ± 1.52	39.61 ± 1.91	< 0.001	42.00 ± 1.42	39.25 ± 1.69	< 0.001
Steep	43.89 ± 1.60	40.96 ± 1.86	< 0.001	43.41 ± 1.53	40.56 ± 1.72	< 0.001
Posterior K (D)						
Flat	−5.98 ± 0.24	−6.01 ± 0.24	0.012	−5.97 ± 0.22	−5.96 ± 0.23	0.218
Steep	−6.38 ± 0.28	−6.43 ± 0.30	0.006	−6.23 ± 1.17	−6.37 ± 0.27	0.094
MPE (μm)	7.46 ± 2.59	8.56 ± 2.97	< 0.001	7.11 ± 2.61	7.86 ± 2.78	0.001
BFSR (mm)						
Anterior	7.88 ± 0.27	8.33 ± 0.34	< 0.001	7.92 ± 0.25	8.43 ± 0.31	< 0.001
Posterior	6.54 ± 0.25	6.53 ± 0.26	0.370	6.57 ± 0.23	6.54 ± 0.25	0.154

*DRS* dual rotating Scheimpflug, *FS-LASIK* femtosecond laser-assisted laser in situ keratomileusis, *SMILE* small incision lenticule extraction, *CCT* central corneal thickness, *ACD* anterior chamber depth, *K* keratometric power, *D* diopters, *MPE* maximal posterior elevation, *BFSR* best-fit sphere radius

*Significant at *p* = 0.01 (=0.05/5), using a Bonferroni adjustment to control for the 5 tests conducted

**Table 4:** Comparison of preoperative and postoperative DRS analyzer differences between the FS-LASIK and SMILE groups

	FS-LASIK	SMILE	*p* value
CCT (*µ*m)	60.86 ± 22.34	66.51 ± 21.01	0.063
ACD (mm)	0.03 ± 0.05	0.07 ± 0.07	< 0.001
Average K (D)			
Anterior	2.69 ± 1.25	2.80 ± 0.98	0.467
Posterior	0.03 ± 0.09	0.03 ± 0.11	0.738
Anterior K (D)			
Flat	2.45 ± 1.40	2.75 ± 0.96	0.076
Steep	2.93 ± 1.23	2.85 ± 1.12	0.634
Posterior K (D)			
Flat	0.02 ± 0.09	−0.01 ± 0.11	0.169
Steep	0.04 ± 0.15	0.17 ± 1.12	0.203
MPE (μm)	−1.10 ± 2.12	−0.75 ± 2.33	0.035
BFS (mm)			
Anterior	−0.44 ± 0.17	−0.50 ± 0.16	0.189
Posterior	0.01 ± 0.05	0.03 ± 0.11	0.225

*DRS* dual rotating Scheimpflug, *FS-LASIK* femtosecond laser-assisted laser in situ keratomileusis, *SMILE* small incision lenticule extraction, *CCT* central corneal thickness, *ACD* anterior chamber depth, *K* keratometric power, *D* diopters, *BFSR* best-fit sphere radius

*Significant at *p* = 0.01 (=0.05/5), using a Bonferroni adjustment to control for the 5 tests conducted

### Corneal Pachymetry

There was no significant preoperative difference in the CCT and spherical equivalent refraction between the two groups. However, the estimated AD or LT were significantly thicker, and the estimated RBT was thinner, in the SMILE group (*p* < 0.001; Table 1).

**Fig. 1:**
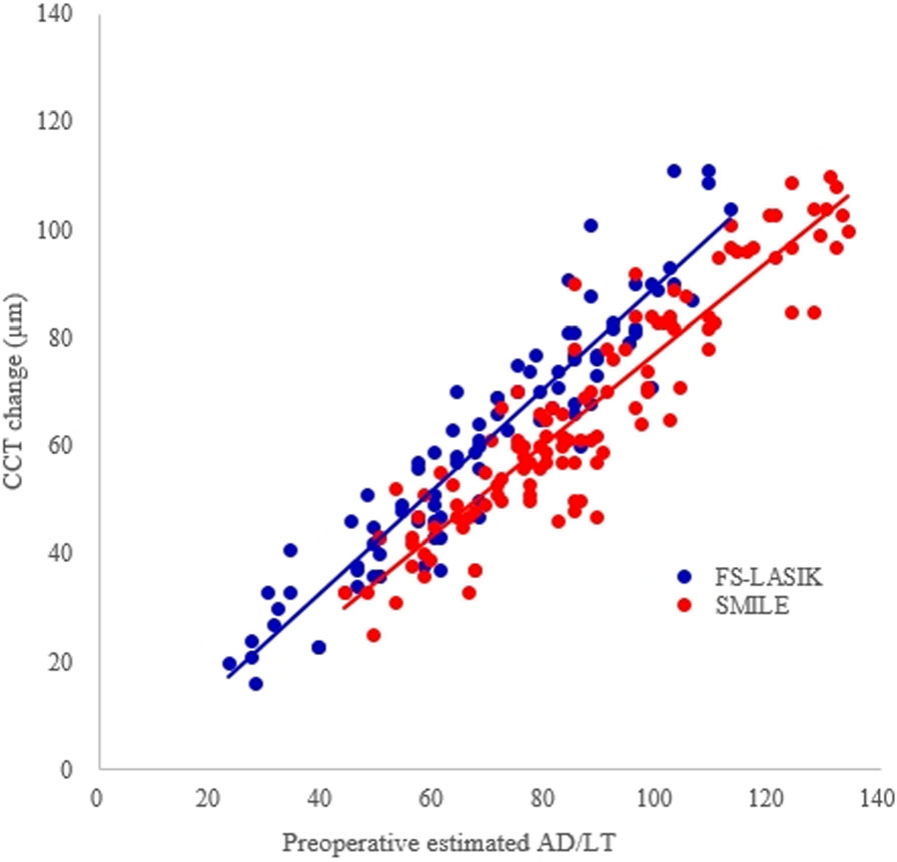
Scatterplots of the correlation between preoperative estimated ablation depth /lenticel thickness versus the central corneal thickness change using the DRS analyzer

Unexpectedly, there was no significant difference between the two groups in postoperative CCT (*p* = 0.367; Table 2) or degree of change in the CCT (*p* = 0.063; Table 4). The mean preoperative estimated AD and LT values were 69.23 ± 22.24 μm and 87.20 ± 22.87 μm, respectively, and the mean change in CCT was 60.86 ± 22.34 μm and 66.51 ± 21.01 μm in the FS-LASIK and SMILE groups, respectively. The percentages of removed corneal thickness were 12.08 ± 4.81% and 13.18 ± 4.38% in the FS-LASIK and SMILE groups, respectively, which showed no significant intergroup difference (*p* = 0.788). There were also significant differences between the preoperative estimated AD or LT and change in CCT in both groups (*p* = 0.011 and < 0.001 in the FS-LASIK and SMILE groups, respectively) (Table 5). The mean difference between the preoperative estimated AD or LT and change in CCT after the procedure was 8.37 ± 7.70 μm in the FS-LASIK group and 20.69 ± 8.73 μm in the SMILE group, which showed a statistically significant difference (*p* < 0.001). The correlation between the preoperative estimated AD or LT and change in CCT after the procedure was high in the SMILE group (*r* = 0.924; *p* < 0.001), but lower than that in the FS-LASIK group (*r* = 0.940; *p* < 0.001; Fig. 1).

**Table 5:** Comparison of the preoperative estimated ablation depth or lenticule thickness and changes in the central corneal thickness

	Estimated AD/LT	Change in CCT	*p* value
FS-LASIK	69.23 ± 22.24	60.86 ± 22.34	0.011
SMILE	87.20 ± 22.87	66.51 ± 21.01	< 0.001

*AD* blation depth, *LT* lenticule thickness, *DRS* dual rotating Scheimpflug, *CCT* central corneal thickness, *FS-LASIK* femtosecond laser-assisted laser in situ keratomileusis, *SMILE* small incision lenticule extraction

### ACD

There was no significant difference in the preoperative or postoperative ACD between the two groups (Table 2). The paired *t-*test showed that the change in ACD after the procedure was statistically significant in both groups (*p* < 0.001; Table 3), and the change in the ACD was significantly greater in the SMILE group (*p* < 0.001; Table 4).

### Anterior and posterior K

Six months after the procedure, statistically significant changes in the flat, steep, and average anterior K were found in both groups (*p* < 0.001; Table 3). There was also a significant change in the steep and average posterior K in the FS-LASIK group (*p* = 0.006 and 0.001, respectively), but not in the SMILE group (*p* = 0.094 and 0.104, respectively; Table 3). However, there was no significant difference after the procedure in the anterior and posterior K differences between the two groups (Table 4).

### Posterior corneal surface displacement

The mean postoperative MPE was significantly increased compared to the preoperative MPE in both groups (*p* < 0.001 and *p* = 0.001 in the FS-LASIK and SMILE groups, respectively; Table 3). The mean postoperative MPE in the FS-LASIK and SMILE groups was 8.56 ± 2.97 μm and 7.86 ± 2.78 μm, respectively; the difference was not significant (*p* = 0.080; Table 2). The mean MPE change was − 1.10 ± 2.12 μm in the FS-LASIK group, and − 0.75 ± 2.33 μm in the SMILE group, without a significant difference in the degree of change in the MPE between the two groups (*p* = 0.035; Table 4).

## Discussion

In this retrospective study of 218 eyes with myopia that underwent FS-LASIK or SMILE, we reported changes in the anterior segment using the DRS analyzer. Both the FS-LASIK and SMILE procedures treat myopia by removing the anterior corneal stroma; however, SMILE preserves the anterior segment of the cornea and is performed using only the FS laser, whereas FS-LASIK requires flap creation and ablation using an excimer laser; thus differences in the degree of change in the cornea after each procedure would be expected [6, 7].

We compared eyes that underwent FS-LASIK with those that underwent SMILE. Notably, there were significant preoperative differences in the ablation depth (AD) or lenticule thickness (LT) and estimated residual bed thickness (RBT) between the two groups, although there was no significant difference in the spherical equivalent refraction or CCT. The estimated LT in the SMILE group was significantly greater than the AD in the FS-LASIK group, although we targeted refraction emmetropia, and all eyes had a spherical equivalent refraction within ±0.75 D at postoperative 6 months. There has been no comprehensive report describing the AD or LT difference between LASIK and SMILE, but some differences have been reported. Lazaridis A et al. [15] compared the corneal clarity and visual outcomes between FS-LASIK and SMILE, reporting that the preoperative mean spherical equivalent refraction did not show a significant difference (− 4.80 ± 2.4 D and − 5.51 ± 1.86 D, respectively; *p* = 0.136), but the mean estimated LT was significantly thicker in the SMILE group (99 ± 38 μm and 116 ± 28 μm, respectively; *p* = 0.017). Wang et al. [16] reported postoperative differences in the degree of corneal biomechanical change between SMILE and LASIK, where the estimated LT was significantly thicker in the SMILE group when the spherical equivalent refraction was less than − 6.0 D even if there was no significant difference in preoperative spherical equivalent refraction and postoperative biomechanical strength of the cornea between the two groups. However, in our study, the difference in the CCT at 6 months after the procedure showed no statistical significance between the two groups (*p* = 0.063; Table 4). The correlation between the preoperative estimated AD or LT and change in CCT after the procedure in the SMILE group was strong, but slightly weaker compared to that in the FS-LASIK group (Fig. 1). Luft N et al. [17] reported discrepancy between preoperative estimation of LT and the decrease in CCT after SMILE using spectral domain-optical coherence tomography. In their study, mean surgical correction of spherical equivalent refraction was − 4.94 ± 1.75 D, and the decrease in CCT was 18.7 ± 5.7 μm smaller than the preoperative estimated LT. This was consistent with our results. When we considered changes in the CCT after a procedure as actual AD or LT, we concluded that the reason for such changes was overestimation of the LT by the Visumax® program; therefore, the method used for LT estimation should be revised after further investigation.

Because the FS-LASIK and SMILE procedures are designed to flatten and weaken the anterior surface of the cornea, a change in the posterior cornea should be expected [18]. Posterior corneal changes are less pronounced than anterior corneal changes after the procedure, because the anterior corneal surface is reshaped directly during the procedure. Meanwhile, the posterior corneal surface may change, but not directly in response to the procedure; rather, the change arises from intraocular pressure and post-operative corneal thinning [19]. Forward displacement of the posterior surface shows similar characteristics to corneal ectasia, and is thought to represent subclinical ectasia [20]. In our study, the maximal posterior elevation (MPE) was significantly increased after the procedure in both groups (Table 3), and the change in the MPE after the procedure was greater in the FS-LASIK group than in the SMILE group even though it was not significant (Table 4). This was consistent with the report by Wang et al. [21], whose study compared postoperative changes in posterior corneal elevation between FS-LASIK and SMILE. In their study, FS-LASIK was associated with a greater increase in posterior corneal elevation than SMILE, as well as a greater reduction in the corneal resistance factor, showing that SMILE caused less weakening of the cornea than did FS-LASIK. This was because SMILE leaves the anterior-most stromal lamellae intact except for the region with the small incision, which acts to stabilize the cornea after the procedure [7].

Because changes in the posterior corneal surface after keratorefractive procedures usually occur, posterior corneal elevation, posterior corneal curvature, posterior asphericity, and posterior K can change [21, 22]. However, because posterior corneal surface changes occur indirectly due to remodeling of the cornea after anterior corneal flattening, changes in the posterior cornea have shown different results [20–24]. Khairat et al. [23] did not observe significant changes in the keratometric power of the posterior cornea after LASIK, but Seitz et al. [21] reported a significant increase in the negative corneal power of the posterior cornea after the LASIK procedure. We observed a statistically significant increase in the negative power of the posterior cornea after FS-LASIK compared to the preoperative results, with no significant change seen after SMILE. This difference may be explained by preservation of a strong Bowman’s layer and the compact anterior corneal stroma in SMILE [16], resulting in more subtle changes in the posterior corneal surface.

Several studies have evaluated changes in the ACD after LASIK and photorefractive keratectomy (PRK) [25–28]. Most studies found that the ACD was significantly decreased after myopic corneal ablation. However, LASIK and PRK do not affect ocular structures other than the cornea, and a decrease in the ACD is inconsistent with forward shifting of the posterior corneal surface after LASIK or PRK. Nawa [29] suggested that the ACD decrease was the result of a decrease in the magnification effect of the cornea after myopic LASIK, and some authors have reported that an ACD decrease was associated with age and accommodation. Nishimura et al. [26] reported that the ACD significantly decreased after myopic LASIK in patients younger than 40 years, and Wang et al. [27] reported that the crystallin lens thickness increased significantly after LASIK, thus leading to a decrease in the ACD. In our study, the ACD was significantly decreased after both FS-LASIK and SMILE, and the decrease in the ACD after SMILE was significantly greater than that after FS-LASIK. Considering that there was no significant difference in age, preoperative spherical equivalent refraction, or the extent of the postoperative decrease in CCT between the groups, two mechanisms that could explain these findings include a difference in the amount of forward shifting of the cornea, and anterior segment remodeling. Zhang and Wang [30] reported that displacement in the central and peripheral parts of the cornea showed a slight backward and forward shifting tendency, respectively, after PRK. Because we did not investigate regional posterior corneal shifting, we could not conclude that ACD changes were the result of postoperative changes in the posterior cornea. However, because we found that the MPE was more protruded after FS-LASIK compared to SMILE, there may be other differences in the posterior corneal surface specific to the procedure. Rosa et al. [28] suggested that a decrease in the ACD after PRK was related not only to corneal change, but also to anterior segment remodeling. In their study, there was no significant change until 3 months after PRK, but a significant change occurred at between 3 and 6 months postoperatively. The exact mechanism of anterior segment remodeling was not clear; however, this phenomenon could be explained by a continuous change in the anterior segment over time caused by a different postoperative response to intraocular pressure of the newly structured cornea. Because we used the DRS analyzer 6 months after the procedure, we determined that the ACD change could be caused by anterior segment remodeling after FS-LASIK or SMILE, and there could have been a difference in the newly structured cornea according to the procedure that was applied.

## Conclusions

Using the DRS analyzer, we investigated differences between the results of FS-LASIK and SMILE and found a significant change in the MPE after both procedures, with the amount of elevation being higher after FS-LASIK than after SMILE even though it was not statistically significant. Also, the steep and average posterior K was significantly changed after FS-LASIK but not after SMILE. These findings are assumed to be due to preservation of the anterior cornea during the SMILE procedure, which is related to biomechanical stability. The degree of CCT thinning after the procedure did not show a significant difference by procedure, although the preoperative LT estimated using the Visumax® program was significantly thicker than the AD estimated using the Mel 90® program. The reason for this discrepancy was overestimation of the LT using the Visumax® program, so this needs to be revised through further investigation. Finally, the ACD was significantly decreased after both procedures, but the degree of decrease in the ACD was significantly greater using SMILE than using FS-LASIK. Possible mechanisms to explain this difference involve forward shifting of the cornea or anterior segment remodeling, although further studies are still needed to definitively establish the reasons for the difference.

## Data Availability

All the data supporting the findings was contained within the manuscript.

## Abbreviations

ACD: anterior chamber depth
AD: ablation depth
BFS: best fit sphere
CCT: central corneal thickness
CDVA: corrected distance visual acuity
D: diopter
DRS: dual rotating Scheimpflug
FS: femtosecond
K: keratometry
LASIK: laser-assisted in situ keratomileusis
logMAR: logarithm of the minimum angle of resolution
LT: lenticule thickness
MPE: maximal posterior elevation
PRK: photorefractive keratectomy
RBT: residual bed thickness
SMILE: Small incision lenticule extraction
UDVA: uncorrected distance visual acuity
